# Potential of Telomerase in Age-Related Macular Degeneration—Involvement of Senescence, DNA Damage Response and Autophagy and a Key Role of PGC-1α

**DOI:** 10.3390/ijms22137194

**Published:** 2021-07-03

**Authors:** Janusz Blasiak, Joanna Szczepanska, Michal Fila, Elzbieta Pawlowska, Kai Kaarniranta

**Affiliations:** 1 Department of Molecular Genetics, Faculty of Biology and Environmental Protection, University of Lodz, Pomorska 141/143, 90-236 Lodz, Poland; 2 Department of Pediatric Dentistry, Medical University of Lodz, 92-216 Lodz, Poland; joanna.szczepanska@umed.lodz.pl; 3 Department of Developmental Neurology and Epileptology, Polish Mother’s Memorial Hospital Research Institute, 93-338 Lodz, Poland; michalfila@poczta.onet.pl; 4 Department of Orthodontics, Medical University of Lodz, 92-217 Lodz, Poland; elzbieta.pawlowska@umed.lodz.pl; 5 Department of Ophthalmology, University of Eastern Finland, 70210 Kuopio, Finland; 6 Department of Ophthalmology, Kuopio University Hospital, 70210 Kuopio, Finland

**Keywords:** age-related macular degeneration, AMD, telomerase, hTERT, autophagy, PGC-1α, peroxisome proliferator-activated receptor gamma coactivator 1 alpha, mTORC1, senescence, DNA damage response

## Abstract

Age-related macular degeneration (AMD), the main cause of vision loss in the elderly, is associated with oxidation in the retina cells promoting telomere attrition. Activation of telomerase was reported to improve macular functions in AMD patients. The catalytic subunit of human telomerase (hTERT) may directly interact with proteins important for senescence, DNA damage response, and autophagy, which are impaired in AMD. hTERT interaction with mTORC1 (mTOR (mechanistic target of rapamycin) complex 1) and PINK1 (PTEN-induced kinase 1) activates macroautophagy and mitophagy, respectively, and removes cellular debris accumulated over AMD progression. Ectopic expression of telomerase in retinal pigment epithelium (RPE) cells lengthened telomeres, reduced senescence, and extended their lifespan. These effects provide evidence for the potential of telomerase in AMD therapy. Peroxisome proliferator-activated receptor gamma coactivator 1-alpha (PGC-1α) may be involved in AMD pathogenesis through decreasing oxidative stress and senescence, regulation of vascular endothelial growth factor (VEGF), and improving autophagy. PGC-1α and TERT form an inhibitory positive feedback loop. In conclusion, telomerase activation and its ectopic expression in RPE cells, as well as controlled clinical trials on the effects of telomerase activation in AMD patients, are justified and should be assisted by PGC-1α modulators to increase the therapeutic potential of telomerase in AMD.

## 1. Introduction

Age-related macular degeneration (AMD) is the main cause of legal blindness and vision loss in the elderly in developed countries. It is a complex, multilateral disease with many confirmed or putative factors involved in its etiology, but the mechanism of pathogenic action of these factors is not completely clear. Oxidative stress is likely the mechanism most often associated with AMD, but in fact it is difficult to find a disorder that does not include oxidative stress in its pathogenesis. Moreover, in many cases it is not clear whether oxidative stress belongs to the causes or consequences of AMD. The role of oxidative stress in AMD is frequently linked with the macula environment and continuous generation of reactive oxygen species (ROS), resulting from the high oxygen metabolism of the retina. However, even when combined with aging, oxidative stress cannot be considered as a sole factor in AMD pathogenesis.

Telomeres are complexes of proteins and DNA, largely in single-stranded form with the 5′-TTAGGG-3′ repeated sequence motif. They protect chromosome ends against degradation and fusion but are progressively shortened with each DNA replication round. The high metabolic rate in the retina may be associated with telomere attrition [1]. The high content of guanine in telomeres makes them vulnerable to oxidative damage, but the complex structure of telomeres impedes access of DNA repair proteins to the site of damage. Critically short or dysfunctional telomeres induce senescence, which plays an important role in AMD pathogenesis, but the length of telomeres is not a deciding factor in senescence induction [2,3]. Telomerase, an RNA-dependent DNA polymerase, extends telomeres and is active in frequently dividing cells (reviewed in [4]). Telomerase regulates gene expression independently of its DNA polymerase activity and several transcription factors mediate this regulation [5,6,7].

In this review, we present the potential of telomere dynamics in AMD pathogenesis and the role of telomerase in telomere maintenance in early AMD and its interactions with proteins important for pathways that are frequently impaired in AMD—senescence, DNA damage response (DDR), and autophagy. These interactions are underlined by activities of telomerase that are different from its canonical telomere-maintenance functions. Finally, we show that peroxisome proliferator-activated receptor gamma coactivator 1-alpha (PGC-1α), a protein essential in AMD pathogenesis, may be a master regulator of telomerase.

## 2. Age-Related Macular Degeneration

**Figure 1:**
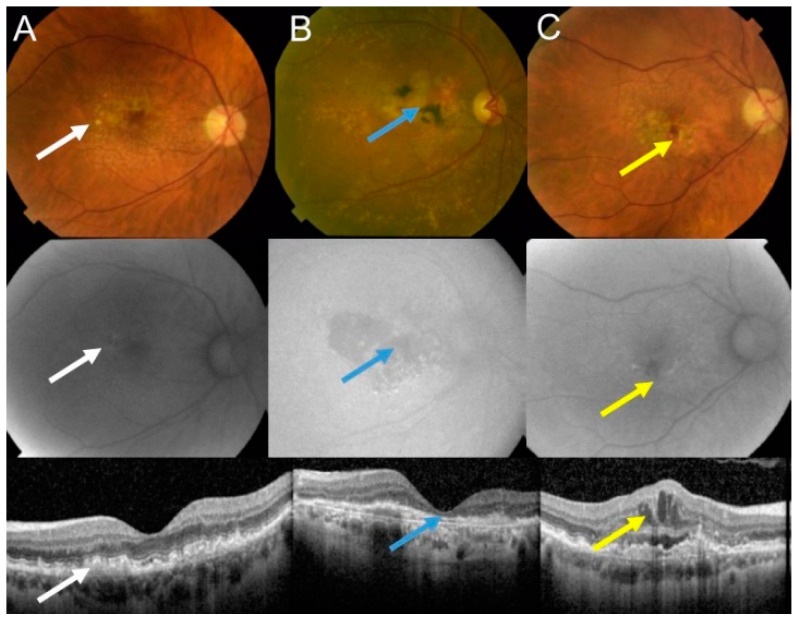
**Age-related macular degeneration (AMD) may basically occur in two forms: dry and wet.** Fundus photograph (upper panel), autofluorescence (middle panel), and optical coherence of intermediate dry AMD with drusen (white arrows) (**A**); advanced geographic atrophy (GA) with typical GA lesions (blue arrows) (**B**); and wet AMD with subretinal and intraretinal fluids and hemorrhage (yellow arrows) (**C**)**.**

The retina is the most energy-demanding tissue in the human body due to the activity of photoreceptors that convert a light pulse into electric signal. Age-related macular degeneration (AMD) is an eye disease affecting the macula, a small structure in the center of the retina containing the fovea, responsible for sharp and color vision. AMD is a serious physical and mental problem for individuals and a high burden for societies [8]. Clinically, AMD is classified into dry (atrophic) and wet (neovascular) forms (Figure 1). Dry AMD is featured by drusen, extracellular white or yellowish objects made of protein and lipids. Vision loss occurs most frequently in the advanced dry form of AMD, geographic atrophy (GA), which impairs photoreceptors and eventually causes their death along with atrophy of the underlying retinal pigment epithelium (RPE) cells [9,10].

The pathogenesis of AMD is not completely known and many factors, both genetic/epigenetic and environmental/lifestyle-based, are involved. Oxidative stress is associated with AMD, but it is not exactly known what the source of such stress is and, in some cases, whether it belongs to the causes or consequences of the disease. Furthermore, oxidative stress can be linked with several putative or established AMD risk factors, including aging, smoking, blue light, obesity, and a diet rich in fat and carbohydrates [11,12,13]. The retina belongs to the most metabolically active tissues in humans with the highest oxygen consumption, resulting in the production of reactive oxygen species (ROS) as byproducts of retinal metabolism [14]. Likewise, aging, as per its definition as the main AMD risk factor, is associated with oxidative stress [15,16]. Also, mitochondria, the main source of energy and ROS production, may be central for AMD pathogenesis [17].

Almost all, if not all, AMD risk factors can directly or indirectly induce DNA damage. Enhanced oxidative stress in the retina can be associated with increased extent of DNA damage in the retinal cells, mainly due to ROS overproduction in these cells.

The retina is provided with nutrients and oxygen by blood flow in two different vascular systems: the central retinal artery system (inner retina) and the choriocapillaris system (outer retina). Blood vessels of the inner retina branch into three capillary layers on entering the eye through the optical nerve [18]. Retinal blood vessels along with ganglion and glial cells form the neurovascular unit, which modulates vessel vasodilatation and vasocontriction, the main regulatory mechanisms of the retinal circulation [19]. As retinal vasculature is a part of the visual system, retinal blood vessels must be transparent, which implies low density of the vessel walls and, in consequence, their high fragility. The retinal vascular endothelium is formed by squamous cells lining the internal surface of retinal blood vessels.

Choroidal neovascularization (CNV) is an essential process in the pathogenesis of wet AMD, which may lead to permanent vision loss if not treated [20]. CNV is featured by abnormal intravasation of choroidal vessels into the RPE or subretinal tissue, which usually leaks and accumulates blood and other fluids in the macula [21]. The new vessels and endothelial disfunction are hallmarks of wet AMD. Vascular endothelial growth factor A (VEGFA) and its receptors are crucial regulators of CNV in wet AMD and are therapeutically targeted in this disease by specific antibodies [22]. The other, most important wet AMD-related angiogenic factor is HIF1A (hypoxia inducible factor 1 subunit alpha) [23]. Therefore, wet AMD is a vascular disease, especially insofar as vascular changes in the form of reduced choriocapillaris density resulting from the loss of endothelial cells may be observed in early stages of disease progression, manifested as a reduction in choriocapillaris density through loss of endothelial cells [20].

Changes in the choroidal vasculature are not only attributed to wet AMD and may vary with disease stage [24]. Eyes with GA displayed a reduced vessel density in choriocapillaris and some of these vessels were hypoperfused [25]. AMD in its early stages is characterized by a low-grade inflammation occurring in the endothelium [26].

Therapeutic options for AMD are essentially limited to its wet form. As mentioned, anti-VEGF therapy is applied in wet AMD cases. Dry AMD, which accounts for about 85% of all AMD cases at diagnosis, has very limited therapeutic options, and these include antioxidant, vitamin, and mineral diet supplementation (reviewed in [27,28,29]). However, the results of these treatments are still ambiguous and studies on new molecular therapeutic targets are warranted. Some experimental, clinical, and conceptual work suggests that telomerase-based therapy might be beneficial in AMD, especially in the dry, largely non-curable form of this disease [30,31,32,33,34,35].

## 3. Telomeres and Telomerase in AMD

Telomeres are DNA–protein complexes at the ends of linear chromosomes in eukaryotes. They protect chromosomes against cellular exonucleases and prevent their recognition as a DNA double-strand break by DNA damage response (DDR) and chromosomal fusion. This is due to masking of the signal of the DNA double-strand break from the chromosomal termini through the six member shelterin complex, a telomere-associated protein, which sequesters telomeric DNA [36]. Each cellular division is associated with shortening telomeres due to the inability of DNA polymerase to completely replicate linear chromosomes—“the end problem” (reviewed in [37]). To protect against deletion in essential genes when the length of telomeres is critically short (crisis), the cell turns to senescence— the mechanism of irreversible inhibition of cellular division and DNA replication. Also Independently of DNA replication, progressive accumulation of oxidative damage in telomeres promotes their premature erosion [38]. However, some cells need to proliferate longer that would follow from the dynamics of their telomeres shortening. One of the mechanisms to overcome the crisis is telomerase, an enzyme whose activity is observed mainly in stem cells, germline cells, and cancer cells, as well as some highly proliferating somatic cells.

Telomerase is an RNA-dependent DNA polymerase, which does not require an exogenous template to synthesize DNA. DNA of human telomeres is largely single-stranded and contains hexameric tandem repeats 5′-TTAGGG-3′. Human telomerase consists of two essential parts: the catalytic subunit (hTERT) and the 451 nt long RNA component (hTERC) containing an 11 nt sequence motif complimentary to telomeric DNA. hTERT uses hTERC as a template to elongate telomeric DNA. Disturbances in telomere biology result in several disorders and some of them are associated with dysfunctional telomerase (reviewed in [39,40]).

RNA-dependent DNA polymerase activity is not the only activity which can be attributed to TERT. Lee et al., using mouse embryonic fibroblasts, neurons, and transgenic mice, showed that TERT promoted cellular and organismal survival independently of its telomerase activity [41]. This independent action was supported by the lack of pro-survival effects of TERC. Under oxidative stress TERT can shuttle from the nucleus to mitochondria where it exerts a protective action on mitochondrial DNA (mtDNA) against various stresses [42,43]. These activities of telomerase are important in the context of AMD pathogenesis, in which oxidative stress and mitochondria play a crucial role [44].

Drigeard Desgarnier et al. analyzed the length of telomeres in different structures of the human eye [45]. They found that neural retina had the longest telomeres, whereas the cornea had the shortest. The length of telomeres in RPE cells was about four times shorter than in the neural retina. These authors did not observed either age-dependent telomere attrition in the retina or any difference in the telomere length between the macula and the rest of the retina.

Immonen et al. did not find any association between telomere length in leukocytes of patients with exudative AMD and large drusen [46]. However, these studies involved only a small fraction (3%) of patients with geographic atrophy—an advanced form of dry AMD.

High content of guanine in telomeric DNA may have at least two consequences. The first is the natural tendency of single-stranded, guanine-rich DNA to fold onto itself to form four-stranded structures due to the ability of guanine to form highly stable hydrogen bonds with other guanines—guanine quartets [47]. This property is important in the context of telomerase action, as four-stranded DNA is not a substrate for that enzyme. The second consequence follows from the fact that guanine has the highest number of oxidation-sensitive sites among all DNA bases [48]. Therefore, telomeres may be remarkably prone to oxidative stress, a common factor of AMD pathogenesis. Moreover, damage to single-stranded telomeric DNA is difficult to repair due to the lack of a template for repair synthesis and limited access of repair proteins to damaged DNA, which may be in a high order conformation and tightly associated with telomeric proteins. It was observed that about half of oxidatively-induced DNA damages remained unrepaired in human fibroblast after 19 days, in contrast to other regions of chromosomes that were recovered within 24 h [49]. Persistent DNA damage in telomeres may lead to their dysfunctions. It was shown that telomere dysfunction activated p53, which bound and repressed the promoters of the *PGC-1α* (peroxisome proliferator-activated receptor gamma coactivator 1 alpha) and *PGC-1β* genes, resulting in metabolic decline observed in degenerative states in aging, quiescent tissues [45,50]. PGC-1α is a member of the transcriptional activators of genes involved in energy metabolism (reviewed in [51]). It is central for mitochondrial biogenesis and this implies its potential in aging and pathogenesis of neurodegenerative diseases [52]. Therefore, telomeres can be a sensitive marker for oxidative, age-related pathologies, including AMD.

Bell et al. observed a unique telomere DNA expansion phenotype in the photoreceptor cell layer of human retinas [31]. These changes were restricted to rod cells and not observed in other retinal cells. Such alterations were not observed in infants before the age of 6 months but increased with age in adults. This unique phenotype was positively associated with the retinal pathologies glaucoma and diabetic retinopathy. The authors suggested that the changes they observed were similar to alterations occurring in some central nervous cancer in which telomere length is maintained by a non-telomerase mechanism, alternative lengthening of telomeres (ALT), which is a kind of homology-directed DNA repair [53]. They concluded that the changes in telomere length they observed were associated with aging and retinal pathologies. As the authors did not observe similar changes in the retinas of rodents, cat, dog, sheep, rabbit, pig, and macaque, they concluded that these alterations might be specific to humans.

Recently, Banevicius et al. observed a higher relative telomere length in the peripheral leukocytes of patients with atrophic AMD than in healthy controls [54].

There is no solid evidence unequivocally showing the activity of telomerase in the adult human normal retina, but several reports indicate that it can be activated in both normal and pathological conditions. In addition, many studies show exosomal expression and activation of telomerase in human retinal cells.

In their landmark work, Bodnar et al. showed that the RPE-340 human RPE cell line in an in vitro culture displayed telomere shortening and senescence, but their transfection with vectors encoding hTERT resulted in telomere elongation, reduced expression of β-galactosidase, a senescence and cellular aging marker, and extension of lifespan by at least 20 doublings [55]. The authors observed a close correlation between extension of lifespan and telomerase activity, which, along with the normal karyotype displayed by transfected clones, indicated that stochastic mutagenesis did not account for observed changes. It is worth noting that virtually all control, i.e., hTERT, clones were senescent or nearly senescent, which raises questions about the suitability of human RPE cells cultures in the determination of physiological parameters and their limitations in studies on AMD pathogenesis.

Rambhatla et al. confirmed the study of Bodnar et al. and showed that RPE-30 cells transfected with hTERT expressed RPE-specific proteins and became melanized in the absence of serum [56]. Although seemingly immortalized, these cells retained the ability for terminal differentiation in vitro.

Retinas in non-human organisms, including goldfish and mice, displayed telomerase activity in both normal conditions and oxygen-induced retinopathy [57,58,59].

Park et al. transformed primary human RPE cells with simian virus 40 large antigen and obtained a clone designated VR3 in pre-mortality stage 2 (M2), as proposed by Wright and Shay [33,60]. Then, the cells were transfected with a vector containing the *hTERT* gene and telomerase was expressed either temporarily (ST1) or continuously (ST2) in two transfected sublines. The lifespan of the VR3 clone was extended by about 20 times more than in normal RPE cells, but it entered a second crisis. The telomere length of VR3 decreased compared with normal RPE cells. The ST1 and ST2 clones that expressed both T antigen and telomerase were able to avoid that crisis. The initial telomere lengths of the ST1 and ST2 clones were longer than their normal cells counterpart. ST1 cells entered cell arrest when telomerase expression was terminated, but ST2 proliferated continuously. Altogether, these results indicated that telomerase activation is required to avoid M2 crisis in the human RPE cells and protect them from senescence.

Dow and Harley showed that a small molecule, TA-65 (telomerase activator-65), activated telomerase and improved macular functions in early AMD patients [32]. TA-65 is a natural product derived from the *Astragalus membranaceus* plant, which upregulates telomerase [61]. This compound was administrated orally twice daily for 1 year. Macular functions were tested by micro-perimetry at the start of the study, at 6 months, and at 1 year. The improvement in macular function started to manifest at 6 months of the treatment and lasted 1 year. The authors claimed that the use of AT-65 in AMD was a paradigm for other TAs in other degenerative age-related disorders.

In summary, studies on the association between telomere lengths in AMD performed in non-target tissue, mainly peripheral blood, did not bring consistent results, likely due to the heterogeneous population of AMD patients. Ectopic expression of telomerase in RPE cells improves telomeres, reduces senescence, and extends their lifespan. These effects, along with a report on the improvement of macular function due to an orally administered telomerase activator in early AMD patients, justify further studies on the potential of telomerase in AMD therapy.

## 4. Senescence, DNA Damage Response, and Autophagy May Underline the Involvement of Telomerase in AMD

Reactive oxygen species produced in oxidative stress may damage DNA and other biomolecules, including those important for DDR [62]. Therefore, impaired DDR may contribute to AMD pathogenesis. Furthermore, oxidative stress and ROS induce stress-induced senescence, different from replicative senescence, but with an even worse outcome [2]. As AMD belongs to the category of proteinopathies, disorders in which protein debris are formed, impaired autophagy is associated with AMD, but the mechanisms underlying this association are still incompletely known [63,64]. Telomerase is, per its definition, engaged in preventing replicative senescence and is reported to be involved in DDR and autophagy.

### 4.1. Senescence

There is a direct association between senescence and telomerase—telomerase prevents senescence in proliferating cells, extending their telomeres and protecting the cells against deletion in essential genes. Moreover, telomerase may extend telomeres that are shortened due to oxidative stress or any other stress resulting in telomere-associated DNA or protein damage.

Replicative senescence covers stable arrest of the cell cycle and is activated as a primary response to telomere deprotection and it includes stimulation of the p53-p21^WAF1^ and/or p16^INK4A^-RB signaling pathways [65]. Impairment of cell cycle checkpoints allows the cells to avoid senescence and proliferate with telomeres being progressively shortened. Finally, such cells develop replicative crises, resulting in fusion of critically short telomeres. This leads to death in a vast proportion of cells, but some of them can survive with genomic instability, loss of cell cycle checkpoint control, and upregulated telomere maintenance, either by activation of telomerase or ALT [66].

Matsunaga studied primary cultures of human retinal cells and showed that after about 60 population doublings, only a minor subpopulation (20%) of cells displayed replicative activity, whereas almost 50% cells displayed expression of β-galactosidase, a senescence marker, and had significantly shortened telomeres [67]. The authors suggested that senescence of the RPE cells might play a role in the development of AMD. The death of retinal cells occurs in the final stage of AMD, but clinical symptoms related to vision worsening and eventual loss are seen earlier when the cells become degenerated and dysfunctional [68]. Kozlowski clearly stated that it is not cell death but senescence of RPE cells that is mainly responsible for AMD development [69]. We expanded on this idea, building a model of AMD pathogenesis in which senescence interacts with DNA damage response, mitochondria, and autophagy [70].

**Figure 2:**
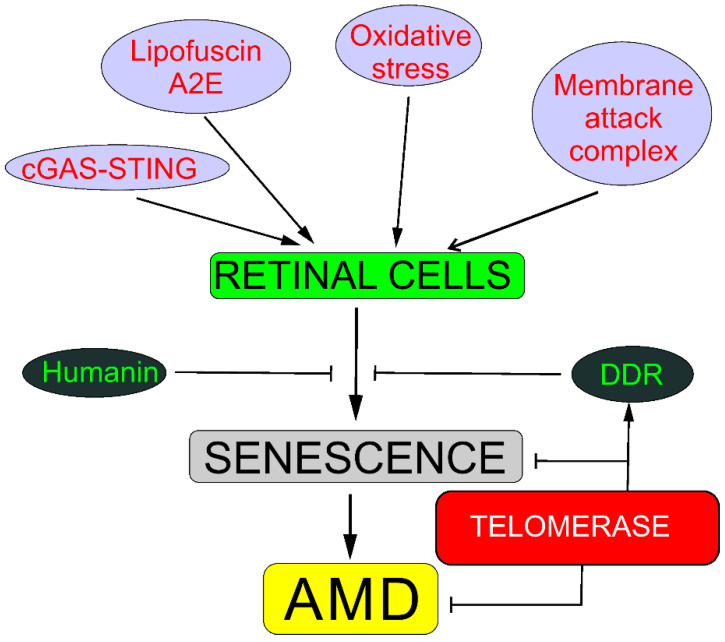
**Telomerase may protect against senescence that may be causal in age-related macular degeneration (AMD).** Oxidative stress evokes stress-induced premature senescence in all kinds of retinal cells, whereas membrane attack complex evokes degeneration of choriocapillaris through senescence in chorioretinal endothelial cells. Lipofuscin and its fluororophore A2E (N-retinylidene-N-retinyl-ethanolamine) show cytotoxic action against retinal cells through mechanisms with the involvement of redox reactions supported by the presence of iron in lipofuscin. Cyclic GMP–AMP synthase (cGAS) detects DNA in the cytosol, leading to the promotion of stimulator of interferon genes (STING), and induces senescence in the response to DNA damage. DNA damage response (DDR) may recover DNA damage, preventing oxidative shortening of telomeres or the activation of the cGAS-STING pathway. Humanin, a mitochondria-encoded peptide, exerts a protective effect against oxidative stress and endoplasmic reticulum stress in human retinal pigment epithelium cells and direct protection against senescence. Different background colors are for better distinguishing different elements.

Senescence may be involved in AMD pathogenesis through several pathways, including oxidative stress response; DDR; humanin; degeneration of choriocapillaris mediated by membrane attack complex; the cyclic GMP-AMP synthase–stimulator of interferon genes (cGAS-STING) pathway; lipofuscin, including its fluorophore A2E (N-retinylidene-N-retinyl-ethanolamine), a byproduct of the visual cycle; and the amyloid-beta peptide (reviewed in [2]). These pathways often overlap. Almost all, if not all, mechanisms of senescence induction in retinal cells are associated with DNA damage. Therefore, DDRs to such senescence-related DNA damage are crucial for senescence occurrence. Telomerase may ameliorate such damage and stimulate DDR, preventing not only replicative senescence but also senescence induced by oxidative stress and, in this way, senescence-related AMD (Figure 2).

### 4.2. DNA Damage Response

Oxidative stress is associated with overproduction of ROS, which may damage cellular macromolecules, including DNA. As mitochondria are the main source of ROS production in the human body, mtDNA is considered to be more prone to damage than its nuclear counterpart (nDNA) in oxidative stress-related pathologies. In fact, several studies show a greater susceptibility of mtDNA than nDNA in AMD (e.g., [71,72,73,74]). Differences in the susceptibility between mtDNA and nDNA to DNA-damaging factors, especially of endogenous origin, in normal conditions are mainly determined by the differences in their metabolism and DDR mechanisms in mitochondria and the nucleus (reviewed in [44]). Moreover, methods used to measure DNA damage in these two organelles are usually different. All mtDNA protein-coding genes specify components of the mitochondrial electron transport chain (mtETC), which produces ROS even in its normal functioning. Damage to genes of the mtETC results in dysfunctional mtETC components, leading to increased ROS production, which may further damage these genes, leading to the accumulation of mtDNA damage—the mitochondrial vicious cycle [75]. Mitochondria also specifically produce reactive aldehydes that can form adducts with mtDNA [76]. Several reports suggest increased levels of DNA damage, both nuclear and mitochondrial, in AMD as well as impairments to its repair, and these effects are mainly attributed to oxidative stress and mutations/polymorphisms of DDR-related genes (reviewed in [62,77]).

Oxidative stress damages telomeres, as their DNA is guanine-rich, and this is why they may be more susceptible to oxidative DNA damage than the “average” DNA in the rest of chromosomes. Honda et al. showed that chronic hyperoxia led to accelerated telomere shortening in RPE-340 cells through an increased accumulation of DNA single-strand breaks in telomeric DNA [78]. These authors obtained similar results for RPE cells that were cryopreserved and thawed, processes inducing DNA damage [79]. In addition, these cells displayed senescence after cryopreservation. As concluded by von Zglinicki, telomere loss due to oxidative damage was in some cases much greater than that resulting from the end replication problem [80]. Furthermore, damage to telomeric DNA may be more difficult to repair due to steric constrains of telomeric DNA in comparison with canonical nucleosomal organization of non-telomeric DNA and other, not fully known, reasons, but some kinds of telomeric DNA are repaired with a higher than average rate [37,81,82]. Therefore, telomerase may extend oxidative stress-shortened telomeres, preventing further degeneration of RPE cells and AMD progression.

**Figure 3:**
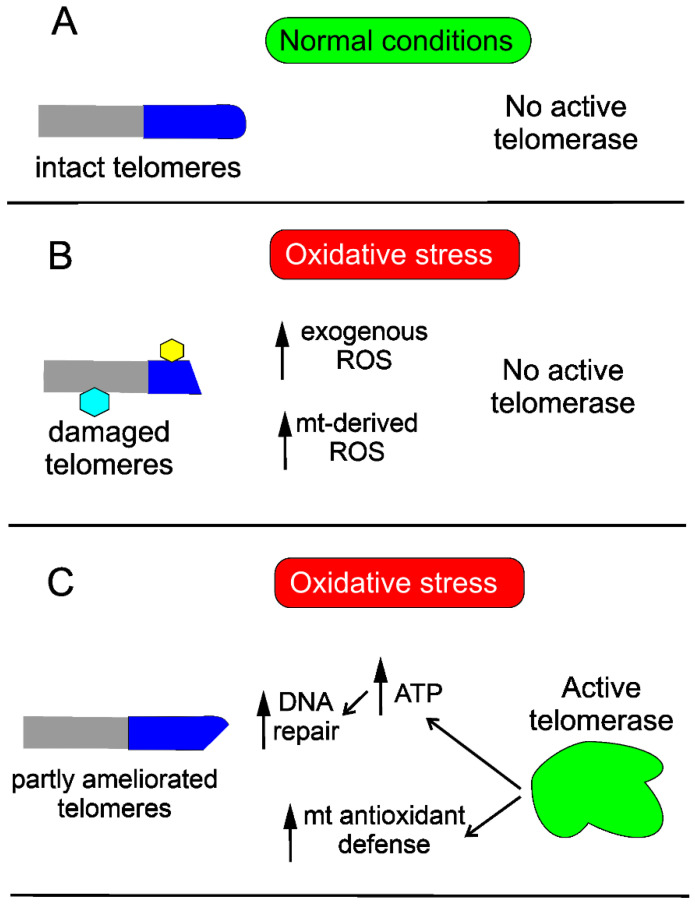
**Telomerase improves telomeres damaged in oxidative stress.** Telomeres (dark blue cap), intact in normal conditions (**A**), may be damaged in oxidative stress (**B**), which leads to stress-induced senescence. Damage to telomeres is presented as their shortening and modification (hexagons), also affecting the non-telomeric part of chromosomes (grey). Oxidative stress may be of endogenous and/or exogenous origin and is associated with reactive oxygen species (ROS) generation that may damage DNA and other macromolecules. Activation of endogenous telomerase or its ectopic expression ameliorates telomeres by improving mitochondrial (mt) antioxidant defense, resulting in decreased ROS levels as well as increasing ATP needed for DNA repair synthesis (**C**). Full recovery of telomeres also requires re-building of the telomere-related protein complex, not presented here.

Telomerase may protect the cell against DNA damage through various mechanisms. Saretzki’s lab showed that overexpression of hTERT in human fibroblasts resulted in a decrease in mtDNA damage induced by oxidative stress [42]. To search for the mechanism underlying the observed changes, they showed that telomerase did not seem to increase the repair of mtDNA damaged by oxidative stress but induced an mitochondrial antioxidant defense mechanism [83]. Altogether, these results show that mitochondrial telomerase may protect nuclear DNA (nDNA) from oxidative stress-induced damage by decreasing mitochondrially produced ROS, which was directly shown for cancer cells [84]. However, it was demonstrated that ectopic expression of hTERT in human primary fibroblasts improved the kinetics of nDNA repair, likely due to an increase in the ATP level [85] (Figure 3).

### 4.3. Autophagy

Nassour et al. showed that cells in replicative crisis displayed an extensive vacuolization, suggesting that autophagy might be involved in the crisis [86]. In fact, they found an increase in several autophagy-related proteins, including lysosomal-associated membrane protein 1 (LAMP1), the autophagy protein 5 (ATG5)–ATG12 conjugate, and LC3 (microtubule-associated protein 1A/1B-light chain 3)-II, while a decrease in the autophagy cargo receptor, p62/SQSTM1 (autophagy receptor p62/sequestosome 1), was observed. The authors concluded that cells in replicative crisis activated autophagy, which resulted in cell death independent of apoptosis. Suppression of autophagy allowed cells to bypass the crisis. Induction of intrachromosomal DNA breaks resulted in apoptosis, whereas DNA damage within telomers activated autophagy. Cytoplasmic DNA resulting from telomeric fusion was necessary for the telomeric autophagic response. Such cytoplasmic DNA triggered the cGAS-STING pathway. In summary, Nassour et al. showed that the elimination of cells in crisis involved autophagy and was essential for genomic stability.

Dysregulated autophagy may contribute to AMD pathogenesis, but its exact mode of action is difficult to define as autophagy is a multifaceted phenomenon, whose final outcome depends on many factors, including the level of cellular and organelle metabolism, DNA damage, proteasomal activity, and other effects [64,70,87,88]. Moreover, autophagy itself raises many unanswered questions and, as Daniel Klionsky said, “participates in, well, just everything” [89].

Although it is not known to what extent oxidative stress in AMD belongs to the reasons or consequences of the diseases, it is reported to be associated with the disease [11,90]. Oxidative stress may result in protein misfolding and unfolded protein response (UPR) to repair misfolded proteins by molecular chaperones, but if this system is not efficient enough, soluble proteins are labeled by ubiquitination and targeted for degradation in the proteasome (reviewed in [91]). The processes of aging and neurodegeneration may affect the efficacy of the proteosomal action, resulting in accumulation of oxidized and ubiquitinated proteins, which, in turn, recruit autophagy receptors such as p62/SQSTM1 and LC3 (reviewed in [91,92]). Disturbed autophagy in RPE cells may lead to the accumulation of lipofuscin and activation of the NLRP3 (NLR family pyrin domain-containing 3) inflammasome, inducing a low-grade chronic inflammation in the retina, typical for AMD [26]. However, Kosmidou et al. asked some important questions concerning the role of NLRP3 in AMD pathogenesis, but these questions have not been addressed so far [93].

Impaired autophagy is associated with lipofuscin accumulation in RPE cells [88,94]. Autophagy may perform pro-life and pro-death cellular functions and their regulation is not completely clear (reviewed in [95]). Mitter et al. suggested that autophagy may be dysregulated in AMD in two ways depending on whether AMD-associated oxidative stress is acute (autophagy increased) or chronic (autophagy reduced) [94]. They concluded that impaired autophagy might increase oxidative stress and in this way play a role in AMD pathogenesis.

hTERT is reported to directly interact with several proteins, including autophagy regulators (reviewed in [96]). Cheng et al. showed that mice with *TERT* or *TERC* knockout displayed a delay in recovery after acute kidney injury [97]. These authors observed an increased formation of autophagosomes in renal tubular epithelial cells in wild-type mice but a delay in their development in knockout mice. Downregulation of LC3 II, prolonged accumulation of p62/SQSTM1, and increased activation of the mTOR (mechanistic target of rapamycin) pathway were also observed in knockout mice. In humans, the mTOR kinase acts in two distinct complexes mTORC1 (mTOR complex 1) and mTORC2. Rapamycin, an mTORC1 inhibitor, partially restored the ischemia/reperfusion-induced autophagic response. Apart from clinical conclusions, this work indicates an interaction between telomerase and mTORC1 contributing to autophagy activation. In a similar study, it was shown that irisin, an energy metabolism hormone, improved autophagy of aged hepatocytes through increasing telomerase activity in hepatic ischemia/reperfusion [98]. It was shown that hTERT inhibited the kinase activity of mTORC1, a negative regulator of autophagy, in several cell lines, resulting in autophagy activation in both normal and amino acid-deprived conditions [99]. Moreover, they observed that cells without hTERT were not able to affect the autophagy flux. In line with this work is the report by Ferrara-Romero et al. who showed that TERC-deficient mice with short telomeres had an overactive mTOR pathway with increased levels of phosphorylated ribosomal S6 protein, a target of mTORC1 [100]. Treatment of TERC-deficient mice with rapamycin decreased their survival, in contrast to lifespan extension in wild-type control animals.

Green et al. found a differential activation of autophagy in normal human fibroblasts expressing hTERT dependent on the subcellular localization of hTERT [101]. Hughes et al. showed that the interaction between telomerase and autophagy was critical for flow-induced human arterial vasodilation [102]. They demonstrated that autophagy acted downstream of telomerase as a common denominator in determining the mechanism of flow-mediated dilation, preventing its switch from nitric oxide to pathological hydrogen peroxide.

Roh et al. showed that hexokinase 2 (HK2), an essential glycolysis enzyme catalyzing the first committed step in glycolysis from glucose to glucose-6-phosphate, can be a molecular link between telomerase and autophagy [103]. They found that the activation of HK2 by telomerase inhibited mTOR activity, leading to autophagy activation. Moreover, these authors noted that telomerase bound to the 5′-TTGGG-3′ sequence in the *HK2* promoter through its RNA component, TERC. The results obtained by these authors suggest that the telomerase-HK2-mTOR-autophagy axis may underline activation of autophagy by telomerase.

Impaired mitochondrial quality control contributes to AMD pathogenesis and disturbed mitophagy may be essential for that contribution (reviewed in [17,104]). Shin et al. showed that hTERT negatively regulated the cleavage and cytosolic processing of PINK1 (PTEN (phosphatase and tensin homolog) induced kinase 1), an essential mitophagy regulatory protein, and supported its mitochondrial localization by inhibiting mitochondrial processing peptidase β (MPPβ) [105]. hTERT promoted mitophagy of dysfunctional mitochondria and improved the functioning of damaged mitochondria through changes in PINK1. In summary, these authors showed that hTERT positively regulated PINK1 function, resulting in increased mitophagy after damage to mitochondria.

The interplay between telomerase and autophagy was shown in several studies on cancer. Ding et al. showed that downregulation of hTERT reduced autophagy and decreased BECN1 (Beclin 1, coiled-coil, moesin-like BCL2-interacting protein) in glioblastoma cells [106]. These cells displayed enhanced levels of ROS and ultimately died. Upregulation of BECN1 or treatment with an antioxidant restored the survival of hTERT knockdown cells. However, telomerase in cancer cells acts specifically on their phenotype and its action cannot be directly extrapolated to normal cells.

**Figure 4:**
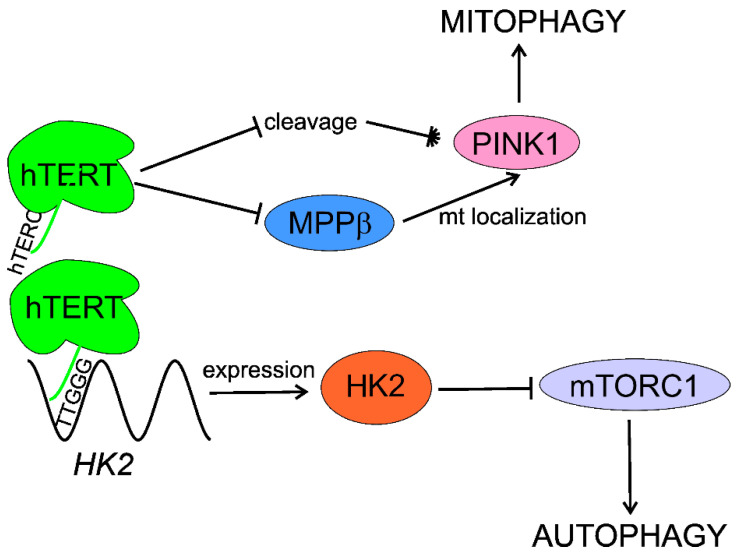
**Telomerase stimulates macroautophagy (autophagy) and mitophagy.** The RNA component of human telomerase (hTERC) binds the 5′-TTGGG-3′ sequence within the promoter of the hexokinase 2 (HK2) gene, stimulating its expression. HK2 inhibits the mechanistic target of rapamycin complex 1 (mTORC1), which is a negative regulator of autophagy. The catalytic subunit of human telomerase (hTERT) negatively regulates cleavage of PINK1 (PTEN (phosphatase and tensin homolog) induced kinase 1) and assists its mitochondrial (mt) localization through inhibition of mitochondrial processing peptidase β (MPPβ), stimulating mitophagy.

In summary, telomerase activates/stimulates autophagy through mTORC1 inhibition that may delay aging and age-related pathologies, including AMD. Telomerase may specifically activate mitophagy by regulation of the PINK1 protein. Although several independent studies show autophagy activation by telomerase, the exact mechanism of this activation is unknown and HK2 can lie between telomerase and autophagy (Figure 4).

## 5. PGC-1α May Link Telomerase with AMD

As previously mentioned, peroxisome proliferator-activated receptor gamma coactivator 1 alpha (PGC-1α) is a member of the group of transcriptional activators of genes involved in energy metabolism (reviewed in [51]). It is central for mitochondrial biogenesis, and this may imply its potential in aging and pathogenesis of neurodegenerative diseases [52]. PGC-1α participates in muscle remodeling and regulation of carbohydrate and lipid metabolism [107]. Except for pathological aging and neurodegenerative disorders, PGC-1α may be implicated in the pathogenesis of several other syndromes, including type 2 diabetes, cardiomyopathy, and obesity [108,109,110].

It was shown that light stimulation of RPE cells enhanced the degradation of photoreceptor outer segments by these cells, which induced the activation of the PGC-1α/ERRα (estrogen receptor related alpha) pathway, which, in turn, upregulated VEGF. Therefore, targeting PGC-1α can be considered in anti-VEGF strategies to increase their efficacy in wet AMD treatment.

Mitochondria are the main source of energy production in cells. This process, even in normal conditions, is associated with the appearance of ROS that may damage biological macromolecules, including DNA. PGC-1α as a regulator of energy production can be also involved in the regulation of ROS production, especially as it is a crucial regulator of mitochondrial biogenesis. It is generally accepted that PGC-1α is an important element of antioxidant defense [111,112,113]. This role is attributed to the regulation of antioxidant enzymes by PGC-1α. However, other mechanisms of the involvement of PGC-1α in the antioxidant action can be considered [114]. In general, the cellular antioxidant system contains three main classes of components: antioxidant enzymes, small molecular weight antioxidants, and DNA repair proteins. As PGC-1α is not an enzyme, it might play a role in DNA repair or more generally in DDR.

**Figure 5:**
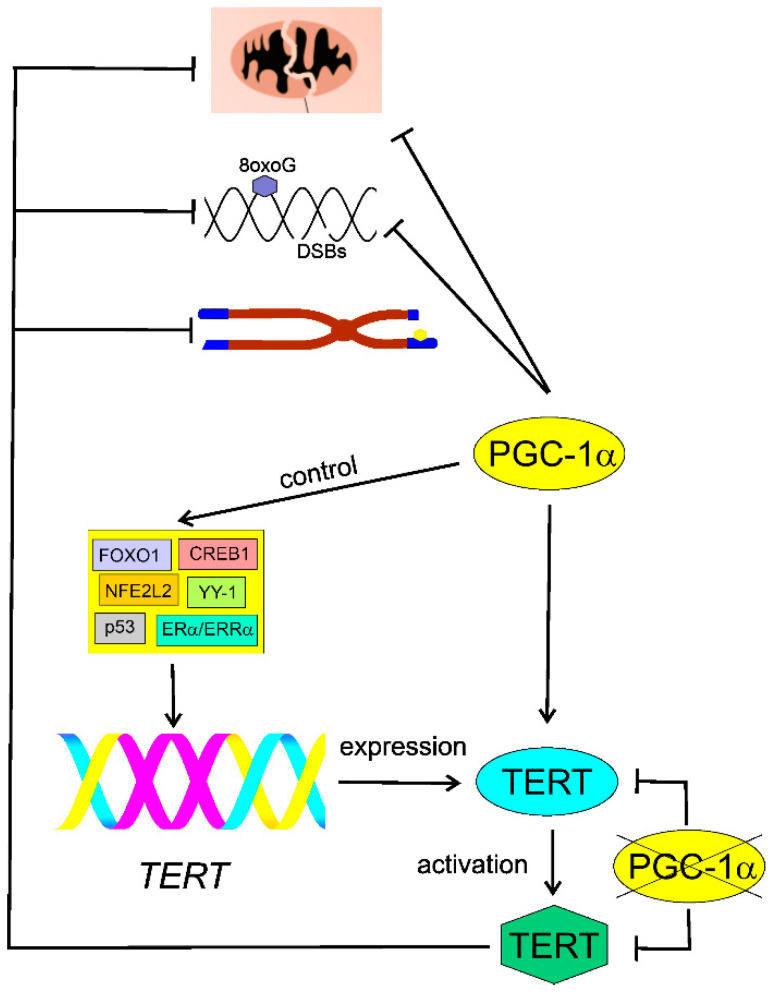
Inhibitory feedback loop between peroxisome proliferator-activated receptor gamma coactivator 1 alpha (PGC-1α) and the catalytic subunit of telomerase (TERT). PGC-1α stimulates TERT, which ameliorates damaged/dysfunctional mitochondria, inhibits DNA damage, and improves shortened or otherwise damaged (yellow hexagon) telomeres (blue boxes) at chromosome (brown) ends. Deletion of PGC-1α results in an increased level of 8-hydroxydeoxyguanosine (8oxoG), a marker of oxidative DNA damage, and other oxidative modifications of DNA bases, as well as DNA strand breaks (DSBs). PGC-1α deletion inhibits expression and activity of telomerase. This effect may be underlined by the loss of control of PGC-1α in transcription factors FOXO1 (forkhead box O1), NFE2L2 (nuclear factor, erythroid 2 like 2), p53, CREB1 (cAMP responsive element binding protein 1), ERα (estrogen receptor 1)/ERRα (estrogen receptor related alpha), and YY-1 (YY1 transcription factor), targeting the promoter (purple) of the *TERT* gene. The lack of PGC-1α (crossed) may inhibit both the expression of the *TERT* gene and its product activation.

Xiong et al. observed an increased extent of 8-hydroxydeoxyguanosine (8oxoG), a marker of oxidative DNA damage, in PGC-1α-deficient aortas of ApoE knockout mice [115]. Moreover, these authors showed increased levels of DNA damage in an alkaline version of the comet assay. As the assay without modification does not detect 8oxoG, the authors speculated that PGC-1α might be involved in the protection against DNA base modifications and strand breaks. However, the alkaline comet assay detects single- and double-strand breaks as well as alkali labile sites. Therefore, a positive output from this technique does not necessarily mean that it is evoked by DNA strand breaks, as these may result from conversion of alkali labile sites in alkaline conditions. To verify whether strand breaks occur, the pH 12.4 version of the comet assay is recommended [116,117]. These authors also observed that PGC-1α stimulated the activity of TERT and ameliorated telomere dysfunction and shortening. PGC-1α deletion decreased the activity and expression of TERT and increased p53 level. These results might evidence a role of PGC-1α in DDR, but the mechanistic basis of this role is not clear. Furthermore, Xiong et al. reported the presence of evolutionarily conserved elements in the TERT promoter targeted by transcription factors controlled by PGC-1α [115]. These were FOXO1 (forkhead box O1), NFE2L2 (nuclear factor, erythroid 2 like 2), p53, CREB1 (cAMP responsive element binding protein 1), ERα (estrogen receptor 1)/ERRα, and YY-1 (YY1 transcription factor) [118]. The main conclusion from the work of Xiong et al. was that PGC-1α and TERT form an inhibitory positive feedback loop and loss of either resulted in loss of the other [119] (Figure 5).

Mitochondrial damage in the RPE may be a trigger for events leading to degradation of RPE and photoreceptors, a hallmark of AMD [120]. Therefore PGC-1α, as a master regulator of mitochondrial biogenesis, is warranted to be studied in AMD pathogenesis (reviewed in [121]). Moreover, PGC-1α also plays a role in the regulation of cellular oxidative stress response, important in AMD [11,24,94,122].

Golestaneh et al. showed that PGC-1α along with SIRT1 (sirtuin 1) were repressed in RPE cells obtained by the dedifferentiation of induced pluripotent stem cells (iPSCs) obtained by reprogramming of skin fibroblasts of AMD and non-AMD donors [123]. The Golestaneh lab showed then that RPE cells obtained from deceased AMD donors displayed upregulated PARP2 (poly(ADP-ribose) polymerase 2), decreased NAD+ (nicotinamide adenine dinucleotide), and dysfunctional AMPK (5′ AMP-activated protein kinase)/SIRT1/PGC-1α pathways as compared with RPE cells obtained from non-AMD eyes. AMD eyes also displayed overactive mTOR pathways [124].

Previously, we genetically modified mice by inactivating mutations in the *PGC-1α* and *NFE2L2* genes [125]. These animals show many characteristics of dry AMD, including age-dependent RPE degeneration, enhanced oxidative stress, impaired mitochondria, changes in protein ubiquitination and autophagy, and vision loss. These changes suggest that the *PGC-1α/NFE2L2* dKO mice can be an animal model of dry AMD.

Saint-Geniez’s lab showed that pharmaceutical upregulation of PGC-1α resulted in a concomitant upregulation of its associated transcriptional factors, antioxidant enzymes, and mitochondrial genes [126]. This was associated with the protection of retinal cell death induced by oxidants. This work provoked Saint-Geniez and her co-workers to check the effect of PGC-1α inactivation in RPE cells. They performed a conditional knock-out in mice and observed that the modified animals displayed the RPE dysfunctions and transdifferentiation associated with degeneration of photoreceptors [127]. They concluded that the changes they observed in the RPE were related to autophagy impairment and epithelial–mesenchymal transition (EMT), both important in AMD pathogenesis. Autophagy can directly regulate EMT by selective degradation of EMT molecular triggers [128]. We partly confirmed Saint-Geniez’s conclusions, showing that cells obtained from *PGC-1α/NFE2L2* dKO aged mice underwent EMT [129]. In that study, we also observed an increased immunoreactivity of senescence markers p16, DEC1 (deleted in esophageal cancer 1), and HMGB1 (high mobility group box protein 1). In their next work, Saint-Geniez and her co-workers showed that induction of EMT in the RPE suppressed *PGC-1a* expression associated with reduced expression of genes involved in the integrity of the mitochondrial network [130].

Ferrington et al. showed that RPE cells obtained from deceased AMD donors were more resistant to oxidative stress than cells obtained from non-AMD controls and the former upregulated PGC-1α in response to the stress [131]. AMD cells had lower respiration and ATP production than controls. This work confirms the protective role of PGC-1α against oxidative stress but it is not in line with many observations showing enhanced oxidative stress in AMD. Moreover, upregulated PGC-1α is rather linked with improvement, and not worsening, of mitochondrial functions [132].

PGC-1α was shown to regulate lysosomal activity of transcription factor E-box binding (TFEB) protein, which is a master regulator of the autophagy lysosomal pathway, in the brains of transgenic mice used as a model of Huntington disease [133]. As TFEB alone reduced protein aggregation, it was concluded that PGC-1α was positioned upstream of TFEB and PGC-1α and that TFEB might have potential in therapies for neurodegenerative proteinopathies. As dysfunctions in protein homeostasis (proteostasis), leading to the accumulation of protein aggregates, are typical for AMD, PGC-1α may hold a promise as a therapy for AMD [63]. Apart from this pro-autophagic effect, PGC-1α reduced oxidative stress and restored mitochondrial function in HD mice.

**Figure 6:**
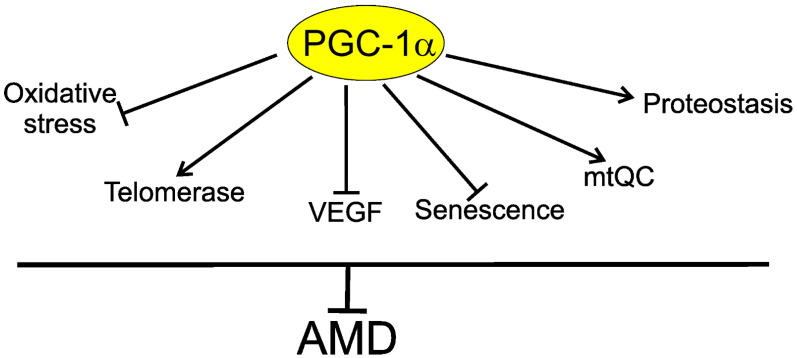
Telomerase is a component of the protective action of peroxisome proliferator-activated receptor gamma coactivator 1 alpha (PGC-1α) against age-related macular degeneration (AMD). PGC-1α may directly or indirectly influence several proteins, mechanisms, and pathways important in AMD pathogenesis, including decreasing oxidative stress, senescence, and vascular endothelial growth factor (VEGF); ameliorating mitochondrial quality control (mtQC) and proteostasis, including autophagy; and stimulating telomerase, which can directly improve impaired telomeres in retinal cells or interact with proteins involved in their protection against factors of AMD pathogenesis.

In summary, PGC-1α may be involved in AMD pathogenesis through several mechanisms, including decreasing oxidative stress in the retina; decreasing senescence of the retinal cells; regulation of vascular endothelial growth factor (VEGF), a key target in wet AMD therapy; and improving disturbed mitochondrial functions and autophagy. PGC-1α and TERT form an inhibitory positive feedback loop and loss of either protein results in loss of the other (Figure 6).

## 6. Conclusions

Short telomeres are associated with several age-related diseases: coronary artery disease, Alzheimer’s disease, chronic obstructive disease, and osteoporosis (reviewed in [134,135]). Shorter telomeres are also observed in some cancers (reviewed in [136]). Stimulation of telomerase expression to alleviate shortened telomeres is of great interest in studies on age-related pathologies (reviewed in [30]). As mentioned, the small, orally administrated compound TA-65, a telomerase activator, improved macular functions in AMD patients [32]. Alpha lipoic acid (ALA), a drug sold in many countries over the counter and reported to reduce atherosclerosis and increase PGC-1α expression in mice, was postulated to increase TERT and ARE/ERE (antioxidant/electrophile-responsive element) signaling to decrease DNA damage and improve telomere functions [115,119,137].

There is not a solid view of telomere length in AMD. In fact, no one has measured the length of telomeres in live human macula yet as there is no method to do so. Several reports indicate alterations in telomere length in peripheral blood cells in AMD patients (e.g., [31,46,54,138]). Since AMD has strong local chorio-retinal characteristics, telomere changes in peripheral blood may not reflect changes in the macula [139,140]. As outlined in this review, some reports suggest a positive association between AMD and telomere length, some a negative one, and others a lack of any association. However, the absolute length of telomeres at a given moment may not be decisive for anything and it is rather telomere homeostasis that matters [3].

Reports on the stimulation of endogenous telomerase and episomal expression of that enzyme suggest that active telomerase in RPE cells may delay degenerative changes in the retina without induction of cancer transformation. This is of therapeutic significance, but the mechanism underlying this effect is not clear. Since telomerase prevents erosion of telomeres and delays the senescence of RPE cells, it may be directly related to AMD pathogenesis [2,67,70,141]. However, as previously mentioned, the absolute length of telomeres might not be causal for cellular states, including senescence [3]. Moreover, most, if not all, RPE cells within the macula are quiescent due to steric constraints and there is no need to compensate for telomere shortening resulting from cellular divisions. This is stress-induced senescence rather than replicative senescence, which contributes to AMD pathogenesis [2]. However, this picture is not completely clear, as RPE cells located in the retinal periphery have more space to divide than their central counterparts and can provide new RPE cells to replace senescent cells within the macula [2]. Therefore, telomerase can be activated to elongate telomeres in some proportion of the retinal cells. However, in the context of AMD pathogenesis and therapy, what seems most important is that hTERT displays activities other than DNA polymerase activity, which are accomplished through its interaction with binding proteins, including proteins important for senescence, DDR, mitochondria maintenance, and autophagy. Telomerase may inhibit mTOR signaling and in this way improve autophagy impaired in AMD. Telomerase interaction with PINK1 may determine modulation of mitophagy.

PGC-1α is reported to play an important role in AMD pathogenesis and to be a potential target in its therapy [121]. There are many pathways underlying these roles of PGC-1α, but it has been shown that it controlled transcription factors specifically targeting the *TERT* promoter [115]. Molecular studies on the ectopic expression of telomerase in retinal cells and controlled clinical trials on the effect of telomerase activation in AMD patients are justified in light of the data showing the involvement of telomerase in many pathways of AMD pathogenesis and promising results for the administration of a telomerase activator in AMD patients. These actions can be assisted by PGC-1α modulators to further explore the role of PGC-1α-TERT interaction and increase the therapeutic potential of telomerase in AMD. However, these perspectives seem to be rational only in early AMD cases, as telomerase only prevents or ameliorates early changes and there is no assumption that its action may reverse advanced degenerative changes in the retina.

## Data Availability

The data that support the findings of this study are available from the corresponding author upon reasonable request.
